# Klippel–Trenaunay syndrome with multiorgan vascular involvement and gastrointestinal bleeding: A case report and literature review

**DOI:** 10.1097/MD.0000000000041634

**Published:** 2025-02-21

**Authors:** Zhihui Wang, Xiaowei Wang, Qing Zhao, Jianli Luan, Yuefeng Ju, Wenzhuo Wang, Feiyue Liu, Shaoting Shi, Shanglang Cai

**Affiliations:** a Department of Gastroenterology, The Affiliated Hospital of Qingdao University, Qingdao University, Shandong, China; b Department of Internal Medicine, The Affiliated Hospital of Qingdao University, Qingdao University, Shandong, China; c Department of Nursing, The Affiliated Hospital of Qingdao University, Qingdao University, Shandong, China.

**Keywords:** diagnosis, endoscopic therapy, gastrointestinal bleeding, Klippel–Trenaunay syndrome

## Abstract

**Rationale:**

To explore the early diagnosis and treatment of Klippel–Trenaunay syndrome (KTS), and provide useful and systematic clinical references for the diagnosis and treatment of such congenital vascular malformations combined with long-term gastrointestinal bleeding.

**Patient concerns:**

A 32-year-old male patient was admitted due to “intermittent rectal bleeding for 32 years, worsening for over 3 months.” The patient had experienced intermittent bright red rectal bleeding since birth.

**Diagnoses:**

After comprehensive clinical examination, imaging evaluation (including abdominal computed tomography and ultrasound), and gastroscopy, the final diagnosis was KTS, and it was found that gastrointestinal bleeding was caused by intestinal vascular malformation.

**Interventions:**

After admission, the patient was provided with gastric acid inhibition and medication for hemostasis. Following the exclusion of contraindications, a colonoscopy indicated the presence of multiple venous varicosities from the splenic flexure to the rectum. Multiple injections of polidocanol and meilan, totaling 45 mL, were administered, leading to significant sclerosis of the varicose vessels. Six months after discharge, endoscopic sclerotherapy for multiple colonic varices was performed again, with a total injection of 40 mL of polydocanol and meglumine.

**Outcomes:**

Throughout the first hospitalization period, there was no recurrence of rectal bleeding. The patient had outpatient follow-up visits after discharge, and the results of routine blood tests showed stable hemoglobin levels. The patient was regularly followed up by telephone after secondary treatment. As of March 2024, the patient had not experienced rectal bleeding and had largely resumed normal work and life activities.

**Lessons:**

Endoscopic therapy can provide significant benefits for patients with KTS complicated by long-term gastrointestinal bleeding. However, KTS cannot currently be cured, and early diagnosis, standardized evaluation, and regular follow-up are key to the collaborative management and treatment of KTS patients.

## 1. Introduction

Klippel–Trenaunay syndrome (KTS) is a rare congenital vascular malformation syndrome. It is characterized by the more common occurrence of isolated superficial venous malformation. Clinically, it is recognized by the presence of port-wine stain, ectatic superficial veins with normal or absent deep veins, and limb hypertrophy triad.^[1,2]^ In 1900, the first 2 cases were separately described by French physicians Maurice Klippel and Paul Trenaunay.^[3]^ Current research indicates that nearly 100% of KTS patients exhibit cutaneous vascular anomalies,^[4]^ with the lower extremities being more commonly affected than the upper limbs and trunk. Although extremely rare, involvement of visceral organs may lead to internal bleeding caused by visceral vascular malformations.^[5]^ Presently, there is no specific treatment for KTS, and management primarily focuses on providing symptomatic relief.^[6,7]^ This article reports a clinical case of multi-organ vascular involvement and gastrointestinal bleeding in a patient with KTS, who lacked the typical port-wine stain, aiming to provide valuable insights for clinical diagnosis and treatment.

## 2. Materials and methods

Analyze the diagnosis and treatment process of 1 patient with KTS complicated by multi-organ vascular lesions and gastrointestinal bleeding admitted to Qingdao University Affiliated Hospital in 2023. Abdominal ultrasound, Computerized tomography (CT) scan, magnetic resonance imaging and endoscopic diagnosis and treatment methods were used. Continuous follow-up and analysis of the results after treatment were conducted and we review the latest KTS-related literature in current databases.

## 3. Results

### 3.1 Basic information

A 32-year-old male patient was admitted to the Department of Gastroenterology at Qingdao University Affiliated Hospital on February 13, 2023, due to “intermittent rectal bleeding for 32 years, worsening for over 3 months.” The patient had experienced intermittent bright red rectal bleeding since birth, occurring about once a day with a volume of approximately 20 mL per episode when bleeding was severe. There were no other associated symptoms. Around 20 years ago, the patient also had urinary bleeding along with rectal bleeding and received intervention for hemostasis at a local hospital, resulting in improvement. However, 3 months prior to admission, the rectal bleeding worsened with a daily volume of about 40 to 50 mL. The patient sought treatment at a local hospital, received red blood cell transfusion and interventional embolization, but the bleeding persisted after discharge. One month before admission, the patient experienced another episode of urinary bleeding, along with dizziness and fatigue. A computerized tomography (CT) scan revealed liver and spleen abnormalities, thickening of the colonic wall, and abnormal signals in the scrotum, pelvic floor, and buttocks. To further diagnose and treat the patient, he was admitted to our department.

The patient is unmarried and has no children. Both parents and 1 sibling are alive. There is no consanguineous marriage between the parents, and the mother had a healthy pregnancy with no history of infection, fever, drug allergies, or trauma. There is no family history of similar or related diseases.

### 3.2 Physical examination

The patient’s complexion, lips, and conjunctiva appear pale. There are no ecchymoses, bruises, or pigmentation on the skin. The abdomen is soft and there is no tenderness or rebound tenderness. The liver is not palpable below the rib cage, and the spleen is mildly enlarged with a firm texture. The right lower limb shows superficial varicose veins and vascular malformations, with more pronounced thickening and swelling compared to the left lower limb, and the skin temperature is slightly elevated upon touch. There are visible varicose veins in the external genitalia, and swelling is observed in the scrotal area and buttocks.

### 3.3 Auxiliary examination

In September 2022, dynamic contrast-enhanced abdominal CT showed multiple slightly low-density lesions in the liver, splenomegaly, and multiple low-density lesions in the spleen, suggesting possible vascular-related abnormalities. Thickening of the rectal wall and multiple peripheral lymph nodes were also observed. There was dilation of the inferior vena cava and left internal iliac vein, along with swelling and edema in the sacral anterior, scrotal, and bilateral buttock subcutaneous areas. Lower limb vascular ultrasound indicated the presence of old thrombosis in the right popliteal vein and muscular venous. Additionally, there were functional abnormalities in the valves of the right femoral vein, great saphenous vein, and small saphenous vein, as well as superficial venous varicosity and swelling in the right lower limb.

**Figure 1.**
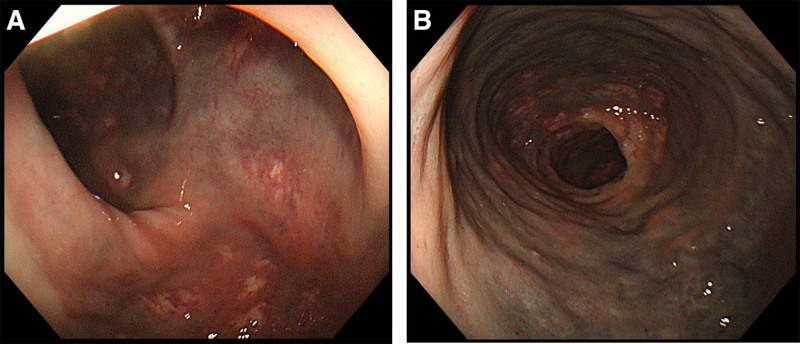
Colonoscopic image demonstrates venous varicosities in the cecum, ascending colon, and transverse colon extending from the anal verge to 50 cm above the anus.

Colonoscopy in November showed venous varicosities in the cecum, ascending colon, and transverse colon extending from the anal verge to 50 cm above the anus (Fig. 1).

**Figure 2.**
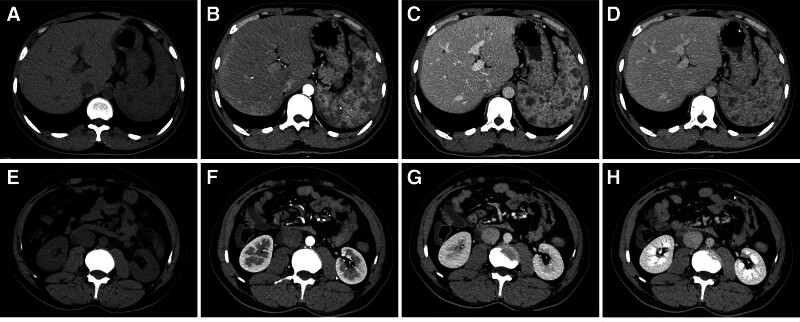
Abdominal dynamic contrast-enhanced CT scan revealed scattered patchy slightly low-density lesions in the liver, splenomegaly, diffuse low-density nodular lesions within the spleen, patchy low-density lesions in the anterior-lateral aspect of the inferior vena cava (below the level of the right renal hilum), retroperitoneal low-density lesion. CT = computed tomography.

**Figure 3.**
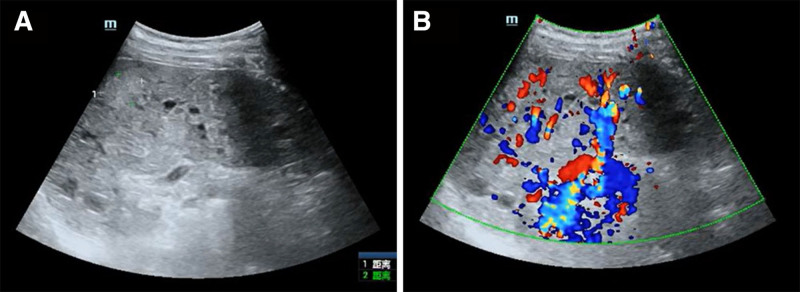
The ultrasound indicates splenomegaly, widespread high-density nodular lesions within the spleen, and a strong likelihood of vascular or lymphatic lesions.

**Figure 4.**
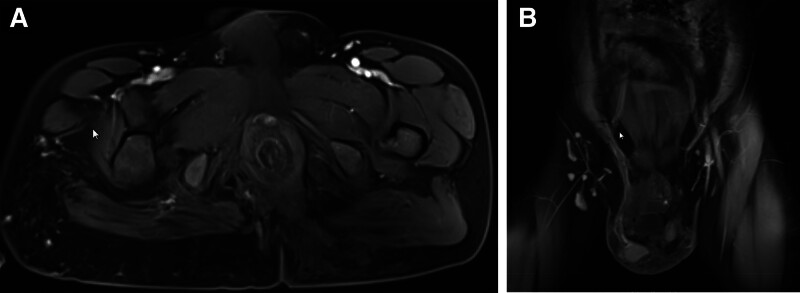
Pelvic MRI revealed rectal wall thickening, mesenteric infiltration, and swelling, along with visible lymphatic dilation. Varicose veins were also noted in the sacral anterior, scrotal region, and both buttocks. Within the scanning range, there was atrophy of the thigh muscles and the muscles in the right buttock, including gluteus maximus and gluteus minimus. MRI = magnetic resonance imaging.

A follow-up abdominal dynamic contrast-enhanced CT scan during this hospitalization revealed scattered patchy slightly low-density lesions in the liver, splenomegaly, diffuse low-density nodular lesions within the spleen, patchy low-density lesions in the anterior-lateral aspect of the inferior vena cava (below the level of the right renal hilum), retroperitoneal low-density lesion (Fig. 2). Combining patient medical history and ultrasound (Fig. 3), there is a strong possibility of vascular lesions. Pelvic magnetic resonance imaging revealed rectal wall thickening, mesenteric infiltration, and swelling, along with visible lymphatic dilation, indicating a possibility of varicose veins. Varicose veins were also noted in the sacral anterior, scrotal region, and both buttocks. Within the scanning range, there was atrophy of the thigh muscles and the muscles in the right buttock, including gluteus maximus and gluteus minimus (Fig. 4).

### 3.4 Treatment process and follow-up

**Figure 5.**
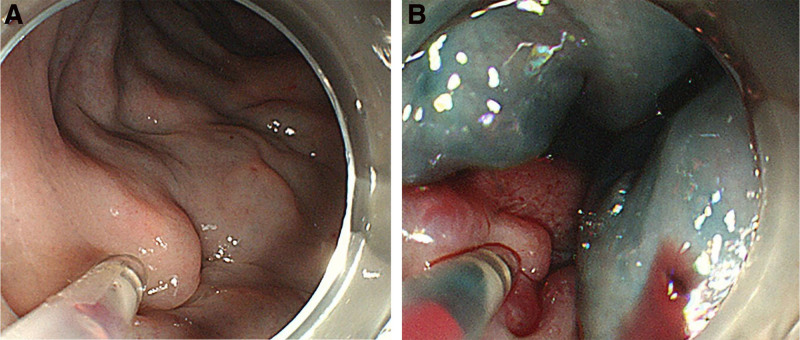
Colonoscopy indicated the presence of multiple venous varicosities from the splenic flexure to the rectum. Multiple injections of polidocanol and meilan, totaling 45 mL, were administered, leading to significant sclerosis of the varicose vessels.

After admission, the patient was provided with symptomatic supportive treatment, including gastric acid inhibition and medication for hemostasis. Following the exclusion of contraindications, a colonoscopy indicated the presence of multiple venous varicosities from the splenic flexure to the rectum. Multiple injections of polidocanol and meilan, totaling 45 mL, were administered, leading to significant sclerosis of the varicose vessels (Fig. 5). After endoscopic treatment, the patient experienced significant pain and was provided with pain relief support. Throughout the hospitalization period, there was no recurrence of rectal bleeding, and a reevaluation of the fecal occult blood test showed a negative result.

**Figure 6.**
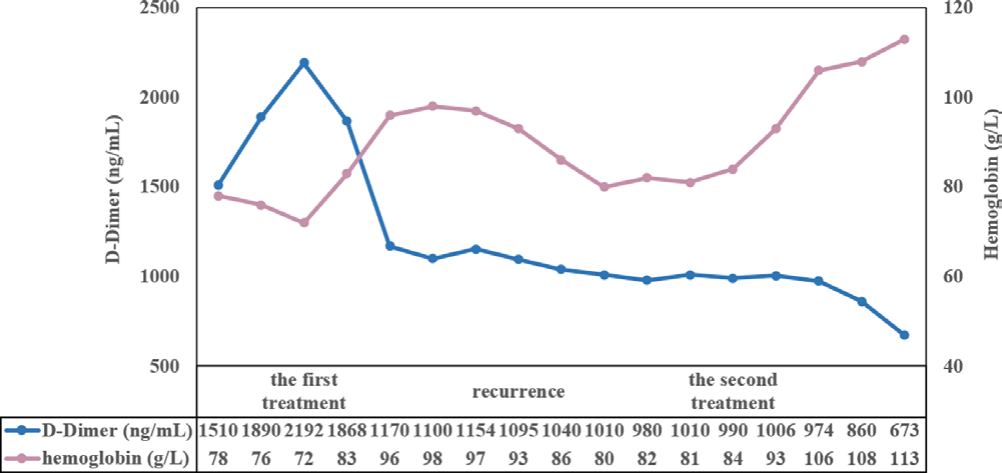
The changes of hemoglobin and D-Dimer during treatment and follow-up.

The patient had outpatient follow-up visits in January and March after discharge, and the results of routine blood tests showed stable hemoglobin levels. Colonoscopy performed in March after discharge revealed multiple colonic varices, and there was no significant bleeding tendency observed after endoscopic sclerotherapy. Six months after discharge, the patient experienced fresh blood in stools again. After colonoscopy, endoscopic sclerotherapy for multiple colonic varices was performed again, with a total injection of 40 mL of polydocanol and meglumine. The patient was regularly followed up by telephone, and as of March 2024, no fresh blood in stools was reported. We also documented changes in key laboratory tests during treatment and follow-up (Fig. 6).

## 4. Discussion

Klippel–Trenaunay syndrome (KTS) is a rare congenital disease characterized by abnormalities in blood vessels and lymphatic vessels, excessive tissue growth, and overgrowth of limbs.^[8]^ It is typically regarded as a benign condition, but due to its multisystem involvement, patients may experience various complications, impacting their quality of life and overall health. There is still no universally accepted gold standard for the diagnosis of KTS.^[9]^ Diagnosis continues to rely on initial assessment based on the patient’s symptoms and signs, supplemented by exclusionary diagnosis through a combination of imaging evaluations, angiography, or genetic testing.^[10–12]^

This report describes a 32-year-old male patient diagnosed with Klippel–Trenaunay syndrome for the first time, presenting with primary symptoms of superficial venous varicosities, swelling in the right lower limb, and vascular abnormalities in the liver and gastrointestinal tract. These multisystem vascular lesions are rare but serious complications of Klippel–Trenaunay syndrome.^[13,14]^ The intermittent gastrointestinal bleeding in the patient is attributed to bleeding from vascular malformations in the gastrointestinal tract. Review of literature indicates that multisystem vascular abnormalities in KTS patients are a relatively rare but documented condition.^[15]^ Among them, gastrointestinal bleeding from vascular malformations is one of the common multisystem complications.According to previous literature reports, the incidence of gastrointestinal bleeding in KTS patients ranges from 2% to 23%, possibly associated with mucosal damage and rupture of small blood vessels caused by vascular malformations. Depending on the patient’s clinical symptoms and severity of bleeding, treatment for gastrointestinal bleeding in KTS patients involves medication,^[16]^ endoscopic therapy, interventional hemostasis, and surgical intervention.^[17,18]^ This patient has underwent 2 sessions of interventional embolization therapy for hemostasis, but the treatment efficacy was not remarkable. Interventional embolization can effectively block bleeding arteries and thus control the source of bleeding. However, due to the vascular abnormalities and variations inherent in KTS patients, interventional methods may have difficulty in fully localizing the bleeding arteries in this case.

The fundamental cause of complications in KTS lies in its extensive vascular malformations, so all treatments for KTS should be based on correcting vascular abnormalities. European vascular surgery guidelines recommend conservative treatment for symptoms and signs secondary to KTS, including limb elevation, compression therapy, and alleviation of congestion through physical methods as much as possible. There are several clinical reports indicating that the condition of most patients can be controlled through conservative treatment measures. For multiple colonic vascular tumors or refractory rectal bleeding, options such as partial colectomy and colorectal-anal anastomosis can be considered.^[14]^ However, upon reviewing relevant published studies, we found that endoscopic therapy has demonstrated significant efficacy in gastrointestinal bleeding associated with KTS. Many case reports and clinical studies indicate that endoscopic therapy can effectively control the source of gastrointestinal bleeding, alleviate bleeding symptoms in patients, and improve their quality of life. Endoscopic technology has significant advantages in detecting occult bleeding sources, implementing hemostasis measures, and monitoring treatment efficacy. Currently, commonly used techniques include hemoclipping, electrocoagulation, laser hemostasis, and sclerosant injection. Sclerosants recommended by European vascular surgery guidelines include ethanol, sodium tetradecyl sulfate, or polidocanol. In reviewing this case, considering the poor efficacy of conservative and interventional embolization treatment, the surgical complications, and their impact on long-term quality of life, we performed a colonoscopy and the first endoscopic treatment for the patient. Colonoscopy revealed multiple venous malformations from the splenic flexure to the rectum, and sclerotherapy was performed with multiple injections of polidocanol and meilan totaling 45 mL, resulting in significant vascular sclerosis immediately after injection. Within 3 months postoperatively, the patient did not experience any rectal bleeding or melena. However, endoscopic treatment still has certain limitations for deep vascular malformations and variations. The patient experienced recurrent symptoms of rectal bleeding 6 months later, although the amount of bleeding was reduced compared to before. We considered the benefits of endoscopic treatment to be significant. With the patient’s consent, the second endoscopic sclerotherapy for rectal varices was performed, and good results were achieved. As of March 2024, the patient had not experienced rectal bleeding, and had largely resumed normal work and life activities.

However, careful discrimination is essential in gastrointestinal endoscopy of KTS syndrome patients, as ulcers might appear on the surfaces of varices or vascular tumors in some cases, potentially leading to misdiagnoses of inflammatory bowel disease. It’s crucial to note that these lesions should not be biopsied, as it may lead to severe bleeding. Instead, the entire gastrointestinal tract should be examined by endoscopy to determine the location and extent of vascular malformations, and appropriate and effective treatment for gastrointestinal bleeding should be provided.^[19]^ Accurate diagnosis serves as the foundation of treatment.

For this patient with concomitant gastrointestinal bleeding, we performed relevant symptomatic treatment through endoscopy. However, the treatment of KTS syndrome should be comprehensive, requiring the cooperation of a multidisciplinary medical team, with the main goals of relieving symptoms, improving patient quality of life, and preventing complications. We conducted screening and management of systemic complications for the patient. Studies have shown that KTS syndrome is associated with an increased risk of thrombosis, possibly due to vascular malformations and localized intravascular coagulation abnormalities, as evidenced by the presence of chronic thrombosis in the right leg’s popliteal and intermuscular veins in this case.^[1]^ Furthermore, we observed significant abnormalities in the patient’s coagulation. In the literature review process, researchers found that KTS can be identified through these diagnostic, including thrombophlebitis, deep vein thrombosis, pulmonary embolism, and congestive heart failure.^[20,21]^ The issues of coagulation and bleeding in KTS patients need to be continuously evaluated and discussed. Considering the extensive gastrointestinal varices and bleeding tendency in this case, we have refrained from anticoagulant therapy for now.

Additionally, regarding the pathogenesis of the disease, studies have suggested that KTS syndrome may be associated with mutations in the PIK3CA gene, which are related to abnormal vascular formation and proliferation, leading to vascular abnormalities and tissue overgrowth in patients. In the patient’s clinical presentation, we also observed significant overgrowth, which aligns with the researchers’ observations.^[22,23]^ Due to the patient’s refusal of genetic testing, we are unable to discuss the pathogenesis of the disease at the genetic level in this case. However, PIK3CA inhibitors as targeted drugs for treating KTS have potential efficacy.^[19]^ These inhibitors can block abnormal vascular formation and proliferation, alleviate vascular and tissue overgrowth, potentially alleviating symptoms and improving disease prognosis in patients, and even reversing vascular remodeling.

In conclusion, Klippel–Trenaunay syndrome is a rare disease, and there are still many uncertainties regarding its etiology, diagnosis, and treatment. When faced with this rare disease, raising awareness among physicians to achieve early diagnosis, standardized evaluation, and regular follow-up is crucial for better management and treatment of KTS patients. Additionally, clinicians should pay close attention to the complications of KTS patients, especially serious symptoms such as gastrointestinal bleeding, and intervene and treat them promptly. This case report aims to enhance clinicians’ understanding of KTS syndrome patients, improve the diagnosis and treatment of their complications, promote awareness of endoscopic diagnosis and treatment for patients with gastrointestinal bleeding, and advance the overall management of this rare disease, with the hope of benefiting such patients more. It provides valuable insights for future research and clinical practice in patients with KTS syndrome complicated by multi-organ vascular lesions and gastrointestinal bleeding.

## Acknowledgments

We acknowledge the contributions of specific colleagues, institutions, or agencies that aided the efforts of the authors, equally for the help of the proofreader and editor.

## Author contributions

**Conceptualization:** Zhihui Wang, Shanglang Cai.

**Data curation:** Zhihui Wang, Yuefeng Ju, Wenzhuo Wang, Feiyue Liu.

**Formal analysis:** Zhihui Wang, Xiaowei Wang, Jianli Luan.

**Investigation:** Zhihui Wang, Qing Zhao, Jianli Luan, Yuefeng Ju, Shaoting Shi.

**Methodology:** Zhihui Wang, Xiaowei Wang, Qing Zhao.

**Resources:** Zhihui Wang.

**Software:** Jianli Luan.

**Writing – original draft:** Zhihui Wang, Xiaowei Wang, Qing Zhao, Jianli Luan, Yuefeng Ju, Wenzhuo Wang, Feiyue Liu, Shaoting Shi, Shanglang Cai.

**Writing – review & editing:** Zhihui Wang, Xiaowei Wang, Qing Zhao, Jianli Luan, Yuefeng Ju, Wenzhuo Wang, Feiyue Liu, Shaoting Shi, Shanglang Cai.

## References

[1] Sharma D Lamba S Pandita A Shastri S. Klippel-trenaunay syndrome - a very rare and interesting syndrome. Clin Med Insights Circ Respir Pulm Med. 2015;9:CCRPM.S21645–4. 10.4137/CCRPM.S21645 PMC4356473 25861232

[2] Alwalid O Makamure J Cheng Q-G. Radiological aspect of klippel-trenaunay syndrome: a case series with review of literature. Curr Med Sci. 2018;38:925–31. 30341531 10.1007/s11596-018-1964-4

[3] Klippel M Trenaunay P. Du naevus variquex osteohypertrophique. Arch Gen Med (Paris). 1990;3:641–72.

[4] Viljoen D Saxe N Pearn J Beighton P. The cutaneous manifestations of the Klippel-Trenaunay-Weber syndrome. Clin Exp Dermatol. 1987;12:12–7. 2820629 10.1111/j.1365-2230.1987.tb01845.x

[5] Samo S Sherid M Husein H Sulaiman S Yungbluth M Vainder JA. Klippel-Trenaunay syndrome causing life-threatening GI bleeding: a case report and review of the literature. Case Rep Gastrointest Med. 2013;2013:813–653. 10.1155/2013/813653 PMC3686071 23862081

[6] Jiang J Lu M Tang X. A young woman with recurrent rectal bleeding. Gastroenterology. 2022;162:1045–7. 34958757 10.1053/j.gastro.2021.12.268

[7] Wang H Lin W Xie C Yang W Zhou J Guo Z. Gastrointestinal involvement in Klippel-Trenaunay syndrome: pathophysiology, evaluation, and management. Orphanet J Rare Dis. 2023;18:288. 37700367 10.1186/s13023-023-02857-5 PMC10496303

[8] Kihiczak GG Meine JG Schwartz RA Janniger CK. Klippel-Trenaunay syndrome: a multisystem disorder possibly resulting from a pathogenic gene for vascular and tissue overgrowth. Int J Dermatol. 2006;45:883–90. 16911369 10.1111/j.1365-4632.2006.02940.x

[9] Oduber CE van der Horst CM Hennekam RC. Klippel-Trenaunay syndrome: diagnostic criteria and hypothesis on etiology. Ann Plast Surg. 2008;60:217–23. 18216519 10.1097/SAP.0b013e318062abc1

[10] Turner VL Kearns C Wattamwar K McKenney AS. Klippel-Trenaunay syndrome. Radiographics. 2022;42:E167–8. 36190869 10.1148/rg.220150

[11] Bertino F Braithwaite KA Hawkins CM. Congenital limb overgrowth syndromes associated with vascular anomalies. Radiographics. 2019;39:491–515. 30844349 10.1148/rg.2019180136

[12] Abdel Razek AAK. Imaging findings of Klippel-Trenaunay syndrome. J Comput Assist Tomogr. 2019;43:786–92. 31609295 10.1097/RCT.0000000000000895

[13] Yilmaz OK Smithson LE. Klippel-Trenaunay syndrome: a case report of a rare vascular disorder identified in a rural Canadian hospital. Rural Remote Health. 2019;19:5348. 31721594 10.22605/RRH5348

[14] Wang H Lin W Guo Z. Klippel-Trenaunay syndrome with gastrointestinal involvement and portal hypertension-evaluation and management. Dig Liver Dis. 2022;54:1455–7. 35527218 10.1016/j.dld.2022.04.005

[15] Harna B Tomar S. Klippel Trenaunay syndrome. Indian J Pediatr. 2020;87:966–7. 32036598 10.1007/s12098-019-03178-x

[16] John PR. Klippel-Trenaunay syndrome. Tech Vasc Interv Radiol. 2019;22:100634. 31864529 10.1016/j.tvir.2019.100634

[17] de la Torre L Carrasco D Mora MA Ramirez J Lopez S. Vascular malformations of the colon in children. J Pediatr Surg. 2002;37:1754–7. 12483649 10.1053/jpsu.2002.36714

[18] Fishman SJ Shamberger RC Fox VL Burrows PE. Endorectal pull-through abates gastrointestinal hemorrhage from colorectal venous malformations. J Pediatr Surg. 2000;35:982–4. 10873049 10.1053/jpsu.2000.6947

[19] Sasaki Y Ishikawa K Hatanaka KC. Targeted next-generation sequencing for detection of PIK3CA mutations in archival tissues from patients with Klippel-Trenaunay syndrome in an Asian population: List the full names and institutional addresses for all authors. Orphanet J Rare Dis. 2023;18:270. 37667289 10.1186/s13023-023-02893-1 PMC10478188

[20] Jafri SZ Bree RL Glazer GM Francis IR Schwab RE. Computed tomography and ultrasound findings in Klippel-Trenaunay syndrome. J Comput Assist Tomogr. 1983;7:457–60. 6302141 10.1097/00004728-198306000-00014

[21] Dubois J Alison M. Vascular anomalies: what a radiologist needs to know. Pediatr Radiol. 2010;40:895–905. 20432007 10.1007/s00247-010-1621-y

[22] Ruggieri M Pavone V Polizzi A Falsaperla R Fichera M Pavone P. Klippel-Trenaunay syndrome in a boy with concomitant ipsilateral overgrowth and undergrowth. Am J Med Genet A. 2014;164A:1262–7. 24478251 10.1002/ajmg.a.36414

[23] Uller W Fishman SJ Alomari AI. Overgrowth syndromes with complex vascular anomalies. Semin Pediatr Surg. 2014;23:208–15. 25241100 10.1053/j.sempedsurg.2014.06.013

